# Measuring the Performance of Visual to Auditory Information Conversion

**DOI:** 10.1371/journal.pone.0063042

**Published:** 2013-05-16

**Authors:** Shern Shiou Tan, Tomás Henrique Bode Maul, Neil Russell Mennie

**Affiliations:** 1 School of Computer Science, The University of Nottingham Malaysia Campus, Semenyih, Selangor, Malaysia; 2 School of Psychology, The University of Nottingham Malaysia Campus, Semenyih, Selangor, Malaysia; McGill University, Canada

## Abstract

**Background:**

Visual to auditory conversion systems have been in existence for several decades. Besides being among the front runners in providing visual capabilities to blind users, the auditory cues generated from image sonification systems are still easier to learn and adapt to compared to other similar techniques. Other advantages include low cost, easy customizability, and universality. However, every system developed so far has its own set of strengths and weaknesses. In order to improve these systems further, we propose an automated and quantitative method to measure the performance of such systems. With these quantitative measurements, it is possible to gauge the relative strengths and weaknesses of different systems and rank the systems accordingly.

**Methodology:**

Performance is measured by both the interpretability and also the information preservation of visual to auditory conversions. Interpretability is measured by computing the correlation of inter image distance (IID) and inter sound distance (ISD) whereas the information preservation is computed by applying Information Theory to measure the entropy of both visual and corresponding auditory signals. These measurements provide a basis and some insights on how the systems work.

**Conclusions:**

With an automated interpretability measure as a standard, more image sonification systems can be developed, compared, and then improved. Even though the measure does not test systems as thoroughly as carefully designed psychological experiments, a quantitative measurement like the one proposed here can compare systems to a certain degree without incurring much cost. Underlying this research is the hope that a major breakthrough in image sonification systems will allow blind users to cost effectively regain enough visual functions to allow them to lead secure and productive lives.

## Introduction

The technology of sensory substitution has a relatively long history of successfully assisting humans with sensory disabilities. A sight-impaired person can recover some visual functionality by learning how to interpret the information relayed by sensory substitution systems. Probably the most successful system in the field of sensory substitution is Braille [1] where the blind can learn how to read through touch. Since then, the field of visual sensory substitution has grown significantly. Many have tried to manipulate haptic devices in order to convey more information to users, e.g.: vibrating devices to indicate obstacles [2], force feedback for the representation of virtual fences [3] and shape visualization through 3D tactile displays [4].

According to the World Health Organization (WHO) [5], there are 285 million people who are visually impaired worldwide with 39 million completely blind. These facts have become the main driving force behind our research (i.e. Luminophonics [6]), where the main aim is to further develop and improve the technology of visual to auditory substitution. In visual to auditory substitution, or image sonification as it is also known, visual information is converted into interpretable sound patterns [7]. Visual to auditory conversion systems are still relevant regardless of technologically advanced techniques such as retinal implants, which are still currently being developed. By applying this method, the blind can partially reconstruct the visual world by interpreting audible soundscapes. Cross-modality conversion between the visual and audio domains has been an active area of scientific research and various multimedia applications [8] attest to this. Examples include vOICe [9], Raster scanning method [8], Hue Music [10], teaching the blind to play video games [11] and recently from our research, Swiping with Luminophonics [6]. As more and more systems are developed, new measurement methods need to be developed in order to evaluate and compare system performances. In the past, there have been several attempts at categorizing and measuring travel aid systems for the blind, including a survey conducted by Dakopoulos and Bourbakis [12]. In spite of this history, there still seems to be no generally accepted standard for performance measurement and comparison.

The lack of universal performance measurements is a concern, especially with the increasing similarity of the systems being invented. As more systems are created, so are different kinds of tests created along with them. There are several problems with these tests. Most importantly these tests are crafted individually to evaluate the features of the system. With these tests, we can know the advantages and disadvantages of the system. For example, SeeColOr invented by Deville et al is very good especially in object manipulation and human navigation [13]. The authors proposed to test their system by introducing experiments that measured how users interacted with the system. It is easy to evaluate the system from the reported experiments but it is not possible to compare it with other systems. There is currently no common experimental method that has been or can be used for all systems.

Although we could in principle compare systems from the results of the above experiments by running the same tests on other systems, but the comparison would not be fair as most of the experiments are specifically catered for SeeColOr’s features. As such, if we would like to compare SeeColOr with the Raster Scanning method by YeoBerger [8], the results would be skewed as the method developed by YeoBerger and SeeColOr do not share a common set of features. E.g. the Raster Scanning method does not support colour features as proposed by SeeColOr. Our group has developed several prototypes based on several different ideas. After conducting several tests, we found that it is hard for us to evaluate the prototypes internally based on the above mentioned experiments. As the prototypes have unique characteristics of their own, some of the tests using human subjects need to be prototype specific. In order to improve the image sonification technique, essentially we need to compare the systems. It is important to make comparisons between systems in order to identify their strengths and weaknesses. Without a standardized performance measurement, it is hard to rank the systems fairly only based on the results of human test subjects.

Another example of psychological experimentation for assessing the performance of sensory substitution systems comes from a team from University of Trier [14], who developed experiments to evaluate the effectiveness of their system on representing colour perception. Proulx MJ and team [15] have also conducted psychological experimentation to examine the effect of learning when using these systems. They have proved that psychological experimentation provides more intrinsic data that can be interpreted (i.e. learning effect and human reactions towards the systems). Although psychological experimentation is ultimately indispensable when evaluating the performance of a system, and would always have to be done, eventually, before deploying the system, it is still a very costly approach (in terms of time and money) for the early exploratory phase of a project. A more suitable approach for the testing, evaluation and filtering of prototypes at the early developmental stage, would have to rely on some kind of mathematical measurement of the conversion process. If this measurement were to moreover avoid certain biases, it could even be instrumental as a universal method for comparing systems. Although it would never replace psychological experimentation, it would allow for cheap and effective prototype exploration and would add some objectivity to the comparison of systems. In this paper, we are proposing a performance measurement that addresses two main issues: information preservation (i.e. How much visual information is preserved in soundscapes?) and interpretability (i.e. How feasible is it for a human user to interpret, or learn how to interpret, the generated soundscapes?).

## Results

**Figure 1:**
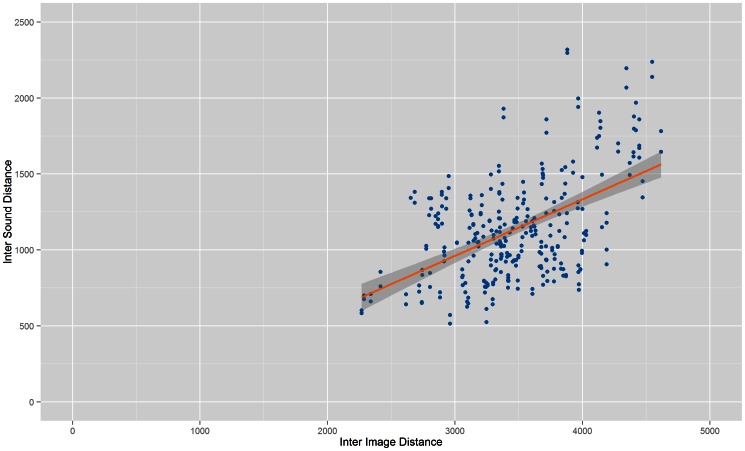
Correlation of Prototype 1. Scatter plot that shows correlation between Inter Image Distance and Inter Sound Distance for Prototype 1.

**Figure 2:**
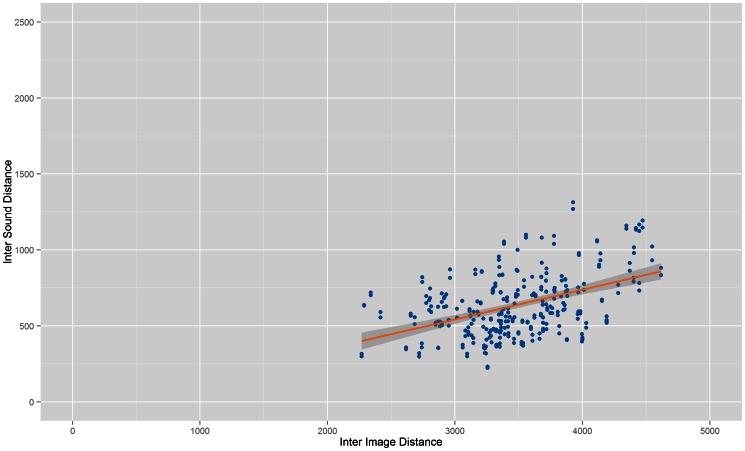
Correlation of Prototype 2. Scatter plot that shows correlation between Inter Image Distance and Inter Sound Distance for Prototype 2.

**Figure 3:**
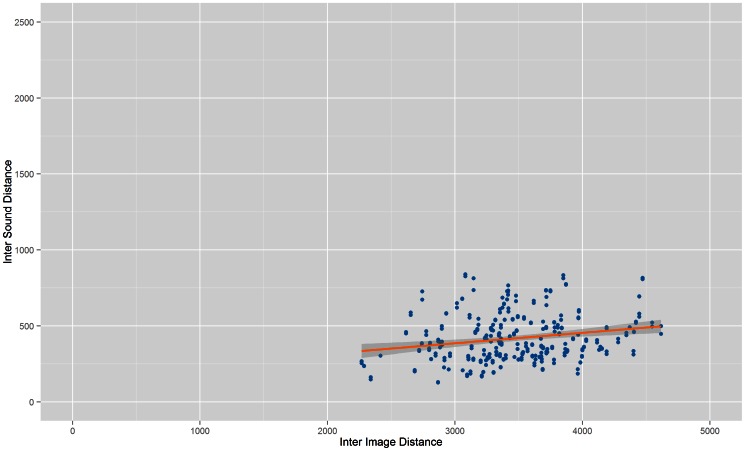
Correlation of Prototype 3. Scatter plot that shows correlation between Inter Image Distance and Inter Sound Distance for Prototype 3.

**Figure 4:**
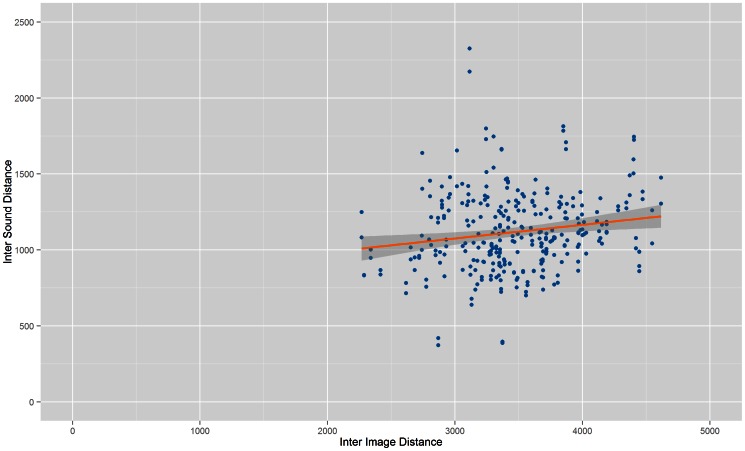
Correlation of vOICe. Scatter plot that shows correlation between Inter Image Distance and Inter Sound Distance for vOICe.

Figure 1 to 4 show the Inter Image Distance (IID)/Inter Sound Distance (ISD) correlation for each system while Table 1 shows the correlation value for each individual system. From the results, Prototype 1 (Table 1) and Prototype 2 (Table 2) have better correlation values compared to Prototype 3 and vOICe. The lowest correlation between Inter Image Distances and Inter Sound Distances belongs to vOICe, i.e.: 0.164991. Both Prototype 3 and vOICe have low correlations probably because they both approach the conversion process with a similar segmentation method whereby the images went through pixelation before being converted into soundscapes.

**Table 1:** Pearson Correlation of IID & ISD.

	Pearson Correlation Value
Prototype 1	0.52087
Prototype 2	0.454596
Prototype 3	0.214192
vOICe	0.164991

**Table 2:** Average Information Lost During Conversion.

	Average Information Lost (%)
Prototype 1	49.359
Prototype 2	51.03441
Prototype 3	45.34453
vOICe	57.55295

As already mentioned, visual to auditory conversions entail dimensionality reduction and therefore information loss is unavoidable. From Table 2, we can see that the conversion process loses about half of the information contained in input images. Our results show that vOICe loses the largest amount of information, which is most likely to be a consequence of it not encoding colour information.

Table 3 summarizes the performance of the systems by ranking them based on the correlation and information preservation. Information preservation and the correlation between IID and ISD are the two main measurement tools presented in this article. We believe that it is better to use both tools rather than one in isolation, because they address different aspects of the quality of the conversion process (information preservation and putative interpretability). As our results show both measurements rank systems differently, and therefore a combined ranking is likely to be more useful.

**Table 3:** Ranking of the Systems.

Systems	Correlation	Information Preservation
Prototype 1	1	2
Prototype 2	2	3
Prototype 3	3	1
vOICe	4	4

## Discussion

In this article, we proposed tools to measure the information preservation and correlation of inter image distances and inter sound distances. These tools are a step towards the accurate and automated prediction of the effectiveness of image sonification systems when adopted by human subjects. These measurements can still be extended and improved. Other measurements relevant to the above mentioned prediction goal should be developed, e.g.: measures of the naturalness of generated soundscapes and estimates of the learning complexity of different conversion processes.

One interesting consequence of having automated and reliable predictors of image sonification performance in human subjects is that we can develop new systems via optimization. If we parameterize image sonification systems in a flexible way (e.g. attentional dynamics, feature extraction steps, etc.), then we can construct cost functions based on the performance predictors (e.g. information preservation and IID/ISD correlation), and run optimization methods (e.g. global stochastic optimization), until we find solutions that maximize performance. Because of the large solution space that can be explored in this cheap and efficient manner we expect very interesting and effective solutions to emerge from this approach.

In the interests of improving the IID/ISD measure it would be useful to conduct a systematic study of what similarity measures are more adequate from the human perceptual point of view. In particular it would be interesting to use human measurements of image or sound similarity and relate these results to automated similarity measures. In this context it would also be pertinent to further investigate the relative suitability of different pre-processing and feature extraction methods.

### Conclusions

The performance measures proposed in this paper are general enough to be suitable for most if not all types of image sonification systems. At their core (if we ignore pre-processing and feature extraction) the measures make no assumptions about the systems and therefore constitute a step towards a fair and efficient means of comparison between systems.

If automated measurements can be applied to rank systems, this will make system testing cheaper (time and money) which will increase the number of proposed systems, and will allow for fair comparisons, which will expedite scientific understanding and help coordinate the research conducted by different groups, which finally will increase the number of technology choices available to the visually impaired. The performance measurements proposed in this article not only serve to measure the effectiveness of existing systems but also serve as a guideline for the improvement of future systems. Even though the metrics proposed can’t be as detailed as the psychological experiments custom built for individual systems, they provide essential information pertaining to the characteristics of image sonification systems.

## Materials and Methods

### Measuring Interpretability

Based on the results from users tested on our prototypes, it was noted that the quality of soundscapes is significantly better with a set of distinctive timbres. To select the most distinctive set of timbres, we decided to measure the inter-soundscape differences between timbres in our library. By generating a distinctive sound signature for each timbre and measuring the differences between these signatures, we could map the differences between all timbres. As already hinted at, this approach became an instrumental stepping stone towards developing a method for measuring interpretability (i.e. correlation between inter image and inter sound distance).

Inter image distance (IID) is the similarity metric between two images. Similarly, inter sound distance (ISD) measures the similarity between two soundscapes. Measuring the relationship between IID and ISD started from the idea of timbre selection. During the process of measuring the distance between audio signals, we found out that there was a strong connection between input image distances and the distances between corresponding output soundscapes and the interpretability of soundscapes. More intuitively, if the soundscapes generated by a system are to have the property of interpretability, then if two images are similar then their corresponding soundscapes should be similar, and conversely if two images are different then their corresponding soundscapes should be different. This property can be easily captured by a correlation measure. This work hypothesizes that the correlation between IIDs and corresponding ISDs, measures to a significant degree the interpretability of the soundscapes generated by image sonification systems.

Research into image similarity measures has been very active in recent decades. With the rise of the Internet, data generated by users has grown exponentially. As a result, search engines continue to develop algorithms for the efficient and accurate retrieval of data. One example of this advancement is the ability to search for images using other images. Partly as a consequence of this a significant number of image similarity measures are being developed and applied for image retrieval purposes.

Although there are several readily available algorithms to choose from, we chose to work with the Earth Mover’s Distance which was first proposed by Peleg, Werman and Rom [16]. The Earth Mover’s Distance (EMD) describes the minimum cost to change a probability distribution to another probability distribution. In our case, a representation scheme was created based on a pair of probability distributions for both images. EMD measures the lowest cost to transform image A to image B with the representation scheme.

In our work, we use an advanced form of EMD for image retrieval by Rubner, Tomasi and Guibas [17]. Instead of using probability distributions, vector quantization is used as the basic representation scheme. This improves the result by taking into account perceptual similarity. Because of this, the approach is well suited for applications that involve colour and texture information. Another reason why Earth Mover’s Distance (EMD) is used for calculating inter image distance is because EMD can be used for calculating the differences between two audio signals. By using the same algorithm for calculating both input (image) differences and output (soundscape) differences, we save on the need of normalizing the results so that they are suitable for each other.

The task of computing inter sound distance between two soundscapes was completed using an existing MATLAB Toolbox to compute music similarity from audio by E.Pampalk [18]. Soundscapes were converted into Mel Frequency Cepstrum Coefficients (MFCC) before using the EMD measurement. MFCC is very important and popular in the domain of sound analysis especially speech processing. MFCC consists of a group of coefficients formed by Mel Frequency Cepstrum (MFC) which is a representation of the sound spectrum on a non-linear mel scale frequency. Most importantly, MFCC records the features of the soundscapes while EMD can compute the distance between two soundscapes through the features in MFCC.

After getting both IIDs and ISDs, a graph can be plotted to show the correlation between the IID and ISD. We proposed to use Pearson Correlation to measure the correlation between IID and ISD with the formula as shown below:-
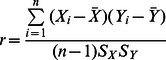
(1)


As already mentioned, we hypothesize that the higher the correlation between IIDs and ISDs is, the more interpretable are the soundscapes generated by a particular system. In other words, we expect that a good image sonification system (i.e. that allows easy interpretation of soundscapes) will exhibit a relatively large IID/ISD correlation.

### Measuring Information Preservation

Converting information from a visual to an auditory form is a process of information reduction. During the conversion, information is lost primarily through dimensional reduction (i.e. conversion of a 2D signal into 1D signal). The degradation of information can be represented by calculating the difference between the entropy of input images and the entropy of corresponding output soundscapes. For the blind to make sense of the soundscapes generated, a sufficient amount of visual information must be preserved in soundscapes.

Najjar suggested in his article Multimedia and Learning [19] that spatial and recognition information are better represented with pictures. The amount of information contained inside a picture allows very rich cognitive encoding that allows high recognition rates. Similarly, Stoneman and Brody [20] found out that children subjected to visual or audiovisual commercial presentations could recognize advertised products more effectively than children subjected only to audio presentations.

In order to make use of the spatial information encoded in an auditory signal, blind individuals need to reconstruct mental images from listening to the soundscapes. For the image sonification to be effective, the reconstructed mental image needs to be sufficiently similar to the real image. Ultimately, although the soundscapes generated by image sonification have gone through severe information reduction, certain aspects of the visual signal such as spatial information need to be retained. Over time, users can learn how to reconstruct the spatial information embedded in soundscapes.

The amount of information preserved needs to be moderately controlled. In most cases, the more visual information that is preserved in soundscapes, the better. With more information, there are more features that can be interpreted by users. For example, if we preserve enough information the user might be able to interpret spatial relationships, shape, colour, shade, texture, motion and so on. However, an excessive amount of information encoded in soundscapes may lead to overwhelming and/or confusing the user.

One of the setbacks of excessive information pertains to difficulties in system adoption. The more visual features are converted into auditory features, the more information needs to be interpreted. This can lead to the problem of cacophony (i.e. confusing mixture of sounds). Moreover, as the number of visual features increases, so does the challenge of finding suitable auditory features to map onto (e.g. what types of sounds should represent which features). As a result, for systems with excessive information, there is a huge learning curve required from users before they can use the system in their daily routines. New users will tend to avoid using the system if it is hard to learn.

On the other hand, systems might lose some of their usefulness if they do not preserve the right amount and type of visual information. For instance, vOICe [9] only encodes grayscale pixels into sound frequency. As a result, it loses colour information during the conversion process, when there might be many situations where colour is essential for decision making.

In conclusion, information preservation needs to strike a balance between sufficiency (too little leads to debilitated decision making) and excessiveness (too much leads to cacophony).

A rough measure of information preservation can be obtained by estimating the “quantity of information” of images and corresponding soundscapes and then calculating the difference between these quantities. For this purpose we make use of a common measure in information theory introduced by Shannon C.E. which is entropy [21]. Entropy is used to measure the unpredictability and uncertainty of a random variable [22]. The easier the variable is to predict, the lower its entropy is. In this case, an assumption is made so that entropy is directly related to the information contained/encoded within the signal. The basic equation for entropy is:-
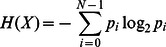
(2)


By computing the entropy of input images and corresponding soundscapes, we get pairs of values that overall represent the information preservation capabilities of particular image sonification algorithms. Because of the dimensionality reduction aspect of the conversion process, the entropy of a soundscape should generally be lower than its image counterpart. Averaging the differences for every matching input (image) and output (soundscape) entropy, we can obtain the relative effectiveness of a system in terms of information preservation. The lower the difference between the entropy of the output and its input, the higher the ability of the system in retaining information during the conversion process. The results of this calculation on several prototypes and an external image sonification system are included and explained in detail in a later section. With this method as a measurement standard, the means for comparing systems in terms of information preservation are made possible.

### Prototypes

The automated measurements as already mentioned were applied to four different systems. The systems include three prototypes from our Luminophonics research and one external system which is vOICe by PBL Meijer [9]. One advantage of this selection is that all of the systems are significantly different from each other. More importantly, the whole conversion process of these systems is well understood. Before we can examine our proposed metrics in the context of the mentioned prototypes, we need to have a clear understanding of how the latter work.

The first Luminophonics prototype is based on swiping as presented in [6]. This was our first attempt to represent colour information via different timbres. Image shapes and objects are converted into segmented blobs. By swiping down from top to bottom, a combination of sounds (with different timbres) is generated. The soundscape produced captures not only colour information but also the vertical position and size of every blob in the image.

The second Luminophonics prototype is a slight departure from the first prototype. Both prototypes include the colour information of blobs, but the swiping method is slightly different in the second case. In the second prototype swiping is also done from top to bottom but different “attentional fields” are provided for the left and right sides of the image. These attentional fields are mapped to the left and right sound channels producing a stereo sound effect capable of providing horizontal positional information. In other words, the horizontal location of blobs is represented using differential volumes in the left/right sound channels. If the blob is situated closer to the middle, the sound of the blob can be heard in both of sound channels with approximately equal volume. Whereas if the blob is situated further to the right side, the sound of the blob can be heard mostly from the right sound channel, and conversely for a blob located mostly on the left side. Prototype 2 makes use of the stereo sound properties of soundscapes to provide information on horizontal positioning.

The third prototype is a combination between the first prototype and traditional frequency-based image sonification methods. Detailed experimentation with the first two prototypes revealed they performed modestly when situations demanded more detailed feature information. This modest performance is due to the blobbing techniques used in both prototypes. Because of this, prototype 3 eliminated the blobbing technique and focused on raw pixels of scaled-down images. Prototype 3 generates a sound from each pixel by swiping from left to right and matching colours to timbres (as with the previous prototypes).

The only external conversion system included in our test was vOICe by PBL Meijer [9]. This approach is simple yet efficient in conveying visual information in the form of soundscapes. Basically, image pixels are converted into different sound frequencies based on their intensity. The sounds generated are then played from left to right (column by column). Although there are other differences, the major distinction between the Luminophonics prototypes and vOICe is that the latter does not consider colour information.
